# Deep Learning-Based Multi-Lead ECG Reconstruction from Lead I with Metadata Integration and Uncertainty Estimation

**DOI:** 10.3390/s26010212

**Published:** 2025-12-29

**Authors:** Ryuichi Nakanishi, Akimasa Hirata, Yoshiki Kubota

**Affiliations:** 1 Department of Electrical and Mechanical Engineering, Nagoya Institute of Technology, Nagoya 466-8555, Japan; r.nakanishi.579@stn.nitech.ac.jp (R.N.); kubota.yoshiki@nitech.ac.jp (Y.K.); 2 Center of Biomedical Physics and Information Technology, Nagoya Institute of Technology, Nagoya 466-8555, Japan

**Keywords:** ECG reconstruction, deep learning, metadata, uncertainty estimation, Monte Carlo dropout, wearable devices

## Abstract

**Highlights:**

**What are the main findings?**

- A dual-branch deep learning model integrating Lead I signals and patient metadata improved 12-lead ECG reconstruction performance.
- Predictive uncertainty estimation using Monte Carlo dropout reflects waveform reliability.

**What are the implications of the main findings?**

- Metadata integration enhances the model performance.
- Uncertainty heatmaps provide interpretable reliability information for clinical use.

**Abstract:**

We propose a dual-branch deep learning framework for reconstructing standard 12-lead electrocardiograms (ECGs) from a single-lead input. The model integrates waveform information from Lead I ECG signals with clinically interpretable metadata to enhance reconstruction fidelity and introduces predictive uncertainty estimation to improve interpretability and reliability. A publicly available dataset of 10,646 ECG records was utilized. The model combined Lead I signals with clinical metadata through two processing branches: a CNN–BiLSTM branch for time-series data and a fully connected branch for metadata. Monte Carlo dropout was applied during inference to generate uncertainty estimates. Reconstruction performance was evaluated using Pearson’s correlation coefficient and root mean square error. Metadata consistently contributed to performance improvements, particularly in the QRS complexes and T-wave segments, and the proposed framework outperformed U-Net when metadata were included. Predictive uncertainty showed moderate to strong positive correlations with reconstruction errors, especially in the chest leads, and heatmaps revealed waveform regions with reduced reliability in arrhythmic and morphologically atypical cases. To the best of our knowledge, this is the first study to incorporate predictive uncertainty into ECG reconstruction. These findings suggest that combining waveform data with metadata and uncertainty quantification offers a promising approach for developing more trustworthy and clinically useful wearable ECG systems.

## 1. Introduction

Electrocardiography (ECG) is one of the most widely employed non-invasive modalities for assessing cardiac electrical activity and remains a cornerstone in the diagnosis and management of cardiovascular diseases—the leading cause of morbidity and mortality worldwide [1,2]. The standard 12-lead ECG provides rich spatiotemporal information, enabling the detection of a broad spectrum of abnormalities, including arrhythmias, conduction disturbances, and ischemic changes [3,4,5]. Despite their indispensable role in clinical practice, the use of full 12-lead systems in ambulatory or out-of-hospital settings is often impractical because of limitations in portability, patient comfort, and electrode placement. To address these challenges, wearable ECG devices, which typically record one or two leads, have been developed and are increasingly utilized for long-term monitoring, telemedicine, and preventive care [6,7,8,9,10]. Although such devices extend monitoring into daily life, their diagnostic coverage remains inherently restricted compared with conventional 12-lead recordings. In practical clinical settings, the primary utility of reconstructed multilead ECGs lies in applications such as triage, remote monitoring, alerting systems, and patient-specific baseline tracking rather than full diagnostic interpretation [11]. These tasks rely mainly on accurate waveform morphology, meaning that high-fidelity reconstruction from reduced-lead recordings remains clinically valuable despite the inherent limitations of wearable devices [12].

A wide range of studies have investigated the reconstruction of 12-lead ECGs from reduced-lead recordings. Classical approaches rely on vectorcardiographic transformations or regression models that exploit linear dependencies among leads [13,14]. With the advent of deep learning, more advanced methods have emerged that can capture nonlinear and temporal dependencies in ECG signals. Convolutional neural networks (CNNs), recurrent neural networks (RNNs), and hybrid autoencoder-based frameworks have demonstrated promising results across diverse datasets [15,16,17]. For instance, Hebiguchi et al. [18] proposed a CNN–BiLSTM hybrid model for reconstructing chest leads from reduced inputs, and Sanjo et al. [19] investigated the sensitivity of electrode placement and waveform distortion to optimize such wearable applications, whereas Savostin et al. [20] and Seo et al. [21] introduced a U-Net–based approach to reconstruct 12-lead ECGs solely from Lead I. In standard ECG acquisition, Lead I represents the electrical potential difference between the left arm (LA) and right arm (RA) electrodes, as defined in Einthoven’s triangle. More recently, Rajotte et al. [22] presented CNN-based models that jointly perform statement classification and lead reconstruction, highlighting the growing trend of integrating diagnostic interpretation with waveform synthesis. More specialized architectures have been introduced to further enhance reconstruction fidelity. For instance, Lence et al. proposed ECGrecover [23], a U-Net-based model designed to reconstruct complete 12-lead signals from incomplete input. Similarly, Manimaran et al. introduced NERULA [24], a dual-pathway framework that leverages self-supervised learning and signal reconstruction to capture detailed cardiac patterns. Additional perspectives on ECG lead reconstruction can be found in earlier review papers [25]. Collectively, these advances underscore the feasibility of reduced-to-multilead reconstruction; however, persistent challenges remain in achieving high fidelity across heterogeneous patient populations and diverse pathological conditions.

A particularly promising but underexplored avenue is the integration of patient metadata as multimodal information. Clinical features such as axis orientation, conduction intervals, and ventricular rate encode physiologically meaningful information complementary to waveform data. Although prior studies have leveraged spatial electrode information, such as lead angles or placement geometry, to improve reconstruction performance [20], the systematic exploitation of broader metadata remains limited to date. In principle, such multimodal integration could improve reconstruction performance; however, in practice, when metadata are restricted to features derived from a single lead, the improvement may be modest. This gap underscores the need to move beyond performance alone and incorporate additional measures of model reliability.

Equally critical is the issue of reliability and interpretability of results. For clinical adoption, it is not sufficient to generate reconstructed waveforms; the reliability of these reconstructions must also be quantified. However, most existing approaches produce deterministic outputs without uncertainty estimates, limiting their interpretability. Bayesian-inspired techniques, such as the Monte Carlo dropout [26,27], offer a practical means to approximate predictive uncertainty, and these methods have been successfully applied in medical imaging and disease detection [28,29,30,31,32]. In the context of ECG reconstruction, uncertainty estimation can generate reliability maps that highlight waveform regions with reduced confidence, such as steep QRS complexes or atypical arrhythmic patterns, thereby supporting clinical decision-making and enhancing trust in AI-based tools.

In this study, we propose a dual-branch deep learning framework that fuses the Lead I signals with patient metadata for 12-lead ECG signal reconstruction. Although metadata integration provides incremental gains in performance, the primary contribution of this study is the incorporation of uncertainty estimation using the Monte Carlo dropout. By providing temporal reliability maps of the reconstructed signals, our framework enables clinicians to identify waveform regions that warrant caution during signal interpretation in monitoring settings. Through comprehensive experiments on a large-scale public database, we demonstrated that metadata integration and uncertainty estimation provided additional information for more interpretable ECG reconstruction, with potential utility in long-term cardiac monitoring and remote triage applications. Unlike prior ECG reconstruction studies that focused primarily on waveform fidelity, our study integrates physiological metadata and predictive uncertainty to achieve interpretable and reliability-aware reconstruction, representing a step toward improved trustworthiness in wearable ECG applications. The primary scope of this study is to enhance the utility of wearable ECG devices for long-term monitoring, remote triage, and preventive screening. Note that the scope of this study does not include regulated medical device design or diagnostic decision-making.

Our framework is not intended to replace standard 12-lead ECGs in acute care or defibrillator systems but to enhance the performance of wearable ECG devices for long-term monitoring. In this context, the main contributions of this study are as follows:

- Propose a dual-branch deep learning framework that reconstructs standard 12-lead ECGs from a strict single-lead (Lead I) input, demonstrating the feasibility of single-lead adaptation for wearable ECG applications.
- Demonstrate that integrating clinically interpretable metadata significantly improves reconstruction performance, particularly in diagnostically important waveform components such as the QRS complex and T wave.
- Introduce predictive uncertainty estimation into ECG reconstruction using Monte Carlo dropout, enabling reliability-aware interpretation that complements conventional accuracy metrics and enhances clinical trustworthiness.

The remainder of this paper is organized as follows. Section 2 describes the dataset, preprocessing, model architecture and evaluation procedures. Section 3 presents the reconstruction results, comparison with U-Net, segment-wise analyses and uncertainty evaluation. Section 4 discusses the implications, limitations and clinical relevance of the study. Section 5 concludes the study.

## 2. Materials and Methods

### 2.1. Dataset and Preprocessing

This study employed 12-lead ECG databases jointly released by Shaoxing People’s Hospital and Chapman University (SPH&CU) [33]. The dataset contained 10,646 patient records, each comprising 12-lead ECGs sampled at 500 Hz for a duration of 10 s. For the reconstruction task, only Lead I was used as the model input, and the remaining 11 leads served as the prediction targets. Diagnostic annotations included normal sinus rhythm, 11 rhythm categories, and 67 disease labels, thereby ensuring sufficient diversity to evaluate the generalizability of the model across both normal and pathological conditions.

Both raw and denoised signals were available; we used the denoised version to reduce the baseline noise and artifacts.

Before being input into the model, all ECG signals were downsampled from 500 Hz to 100 Hz using direct decimation (selecting every 5th sample) to reduce the computational cost and match the receptive field of the network. Because we utilized the denoised version of the dataset, where high-frequency artifacts had already been suppressed, no additional anti-aliasing filter was applied during this step to preserve the QRS sharpness. Each 10-s recording therefore contained 1000 samples per lead at 100 Hz, from which we extracted a fixed 512-sample window (5.12 s) as the model input. This window retains the essential ECG morphology while providing sufficient temporal context for the reconstruction task.

As inputs, we combined Lead I signals with metadata features derived from Lead I: RAxis (ventricular depolarization direction), TAxis (ventricular repolarization direction), Ventricular Rate (beats per minute), QRS Count (number of QRS complexes in 10 s), and QRS Duration (QRS width). Metadata features were discretized into clinically meaningful categories based on the established ECG interpretation guidelines. To ensure that the model captured physiologically meaningful patterns, continuous metadata features were converted into categorical variables, using established clinical standards. Specifically, the thresholds for the RAxis, QRS duration, and ventricular rate were defined in accordance with the AHA/ACCF/HRS recommendations for the standardization and interpretation of the electrocardiogram [1]. The TAxis was categorized using the same angular thresholds as those applied to the RAxis. Furthermore, the QRS Count, which was calculated from the 10-s recordings, was discretized based on its direct mathematical correspondence to heart rate (HeartRate=6×QRS Count). Consequently, the thresholds for the QRS Count were set to align with the standard clinical definitions of bradycardia (<60 bpm), normal sinus rhythm (60–100 bpm), and tachycardia (>100 bpm). The detailed discretization thresholds for all the metadata features are listed in Table 1.

This preprocessing prevents the model from learning inappropriate ordinal relationships while enabling effective modeling of nonlinear interactions among physiological parameters. Importantly, reflecting medically established classification systems is expected to improve both training stability and overall reconstruction performance.

The dataset was randomly divided into training (70%), validation (15%), and testing (15%) subsets. Specifically, the number of records in each subset was 7411 for training, 1588 for validation, and 1588 for testing. Given the large scale of the dataset (over 10,000 subjects), the fixed test set of approximately 1600 records provides a sufficient sample size for reliably estimating the generalization performance. Therefore, a fixed split strategy was adopted instead of the k-fold cross-validation. Moreover, because the dataset contains only one record per patient, no individual can appear in both the training and test sets.

### 2.2. Model Architecture and Training

**Figure 1:**
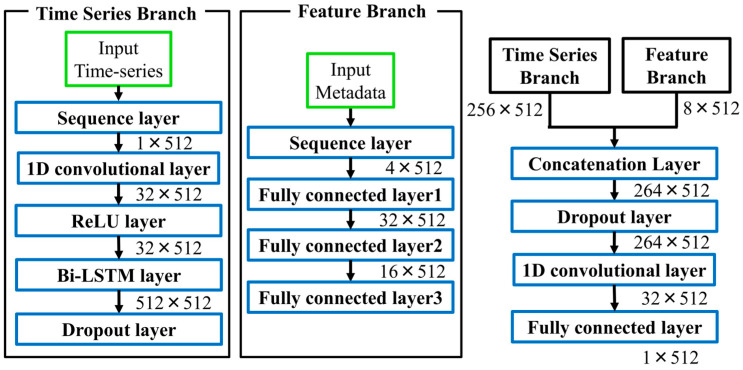
Architecture of the proposed dual-branch ECG reconstruction model. The numerical values next to each block indicate the output tensor shape (Channels × Time steps).

Figure 1 illustrates the overall architecture of the proposed dual-branch model. Lead I waveforms were processed through one-dimensional convolutional (1D-CNN) layers with ReLU activation, extracting local patterns such as P waves, QRS complexes, and T waves, while introducing nonlinearity. The time-series input to the model has a shape of 1 × 512, representing a 5.12-s window of Lead I. Metadata features are converted into categorical variables based on clinical criteria and subsequently one-hot encoded, resulting in a D-dimensional vector (D = number of categories across all metadata attributes). Therefore, the combined input consists of a continuous waveform and discrete metadata vector. These features were then passed to a Bi-directional Long Short-Term Memory (Bi-LSTM) layer, which captures both past and future temporal dependencies, providing a richer sequence representation than conventional unidirectional LSTMs [34,35].

Feature Branch. Patient metadata were input into a three-layer, fully connected network. This design enables the gradual abstraction of nonlinear interactions among clinical features, such as between QRS duration and axis deviation, which may reflect latent cardiac pathophysiology.

Fusion. The outputs of the two branches were integrated using a late fusion strategy and then passed through fully connected layers, allowing the integration of temporal dynamics with metadata-derived abstractions. Such multimodal fusion has been widely recognized as advantageous in biomedical machine learning [36].

The model was trained for 100 epochs with a batch size of 32 using the Adam optimizer [37]. The mean squared error (MSE) loss was minimized during the training process. The training set was shuffled at each epoch, and validation was performed every 20 epochs to monitor the convergence. The model with the lowest validation loss was retained to prevent overfitting. All experiments were performed using MATLAB R2024a.

The hyperparameters were optimized using Bayesian optimization. The search ranges were as follows: BiLSTM units [128–1024], convolutional filters [16–128], kernel size [3–9], dropout rate [0.1–0.3], and learning rate [0.001–0.005]. Bayesian optimization was used to select the optimal configuration based on the lowest validation root mean square error (RMSE). The final model used a two-layer convolutional front-end with 32 filters and a kernel size of 5, followed by a BiLSTM layer with 512 units and a dropout rate of 0.2. The learning rate of the final model was set to 0.001.

### 2.3. Experimental Conditions and Evaluation

To systematically evaluate the contribution of the metadata, three input configurations were tested:

1. Single-feature integration: Each metadata feature was individually added to Lead I.
2. Pairwise integration: Two features that showed superior performance in the single-feature experiments were combined.
3. Full integration: All the metadata features were incorporated.

For benchmarking, the proposed model was compared with a re-implemented U-Net under identical input conditions [20]. The metadata were concatenated along the channel dimension to ensure compatibility with convolutional layers. The comparative conditions included (a) Lead I alone, (b) Lead I plus axis information, and (c) Lead I plus the best-performing metadata combination. For a fair comparison, the U-Net model received the same 512-sample Lead I segment (shape: 1 × 512). When metadata are included, the one-hot encoded metadata vector is repeated along the temporal axis and concatenated with the waveform along the channel dimension, resulting in an input tensor of size (C + 1) × 512. In addition, to assess the contribution of the proposed dual-branch input strategy, a baseline model sharing the same backbone architecture but without the dual-branch design was used for comparison

The reconstruction performance was primarily evaluated using Pearson’s correlation coefficient (R) between the reconstructed and reference signals across the 11 target leads (II–V6). All quantitative evaluations (including Table 2 and Table 3) were performed using the 5.12-s (512 samples) waveforms extracted from the central region of the original recordings. To assess the reconstruction performance of specific cardiac events, we performed a physiological sub-segment analysis (Table 4). First, the R-peaks were detected in the reference signals. Subsequently, individual heartbeats were aligned based on the R-wave location. From these aligned beats, the P-wave, QRS-complex, and T-wave regions were extracted based on standard temporal windows relative to the R peak. The reconstruction accuracy (Pearson’s correlation coefficient) was calculated for each of these physiological segments to determine how well the model reconstructed the distinct parts of the cardiac cycle. Furthermore, for benchmarking, the reconstruction performance was assessed not only using Pearson’s correlation coefficient (R) and RMSE, but also using the structural similarity index (SSIM) to evaluate the morphological consistency between the reconstructed and reference waveforms.

All statistical tests (Wilcoxon signed-rank tests for correlation coefficients (R) and SSIM, paired *t*-tests for RMSE, and Holm correction for multiple comparisons) were performed at a significance level of *p* < 0.05, with significant differences indicated by asterisks.

### 2.4. Uncertainty Visualization and Reliability Assessment

Uncertainty was quantified using Monte Carlo dropout with *N* stochastic forward passes performed during inference [26,27]. Unlike conventional deterministic inference, the dropout layers located after the Bi-LSTM block and the concatenation block, both configured with a dropout rate of 0.2, remained active during testing, and *N* = 1000 stochastic forward passes were performed. The predictive standard deviation *σ*(*t*) at each time point was calculated as follows:

(1) $$\sigma \left(t\right)=\sqrt{\frac{1}{N}\sum_{i=1}^{N}(y_{i}(t)-\bar{y}(t){)}^{2}}$$

where $y_{i}\left(t\right)$ is the *i*-th prediction and $\bar{y}\left(t\right)$ denotes the mean prediction. Because the scale of $\sigma \left(t\right)$ varied across patients, a normalized standard deviation was defined to enable inter-subject comparison:

(2) $$\sigma_{norm}\left(t\right)=\frac{\sigma (t)}{{MAX}_{t}\sigma (t)}$$

To examine whether the predictive variance reflects the reconstruction fidelity, the relative error *e*(*t*) was computed as

(3) $$e(t)=\frac{\left|y_{pred}(t)-y_{true}(t)\right|}{\left|y_{true}(t)+\varepsilon \right|}$$

where ε is a small constant introduced to avoid numerical instability.

Finally, the correlations between $\sigma_{norm}\left(t\right)$ and $e\left(t\right)$ were analyzed across all test cases (*n* = 1588). This analysis verified whether the variance estimates from the Monte Carlo dropout served as meaningful reliability indicators.

## 3. Results

### 3.1. Impact of Metadata on Reconstruction Performance

Table 2 compares the reconstruction performance in terms of Pearson’s correlation coefficient (R) and root mean squared error (RMSE) when using Lead I alone versus the combination of Lead I and individual metadata features. Incorporating metadata consistently improved the reconstruction performance across nearly all leads. Notably, the axis orientation and conduction-related parameters yielded the largest gains in both correlation and error reduction, indicating that physiologically interpretable metadata provide additional predictive value and signal fidelity beyond single-lead input.

Table 3 presents the reconstruction performance for the combination of multiple metadata features. Pairwise combinations significantly improved the performance beyond single-feature integration, and the simultaneous use of all metadata features achieved the highest correlation coefficient and the lowest RMSE among all conditions. Statistical testing confirmed that these improvements were significant (*p* < 0.05) compared to the baseline. Although the overall magnitude of improvement was moderate, the results clearly demonstrated that metadata integration consistently and meaningfully contributes to ECG reconstruction performance.

**Figure 2:**
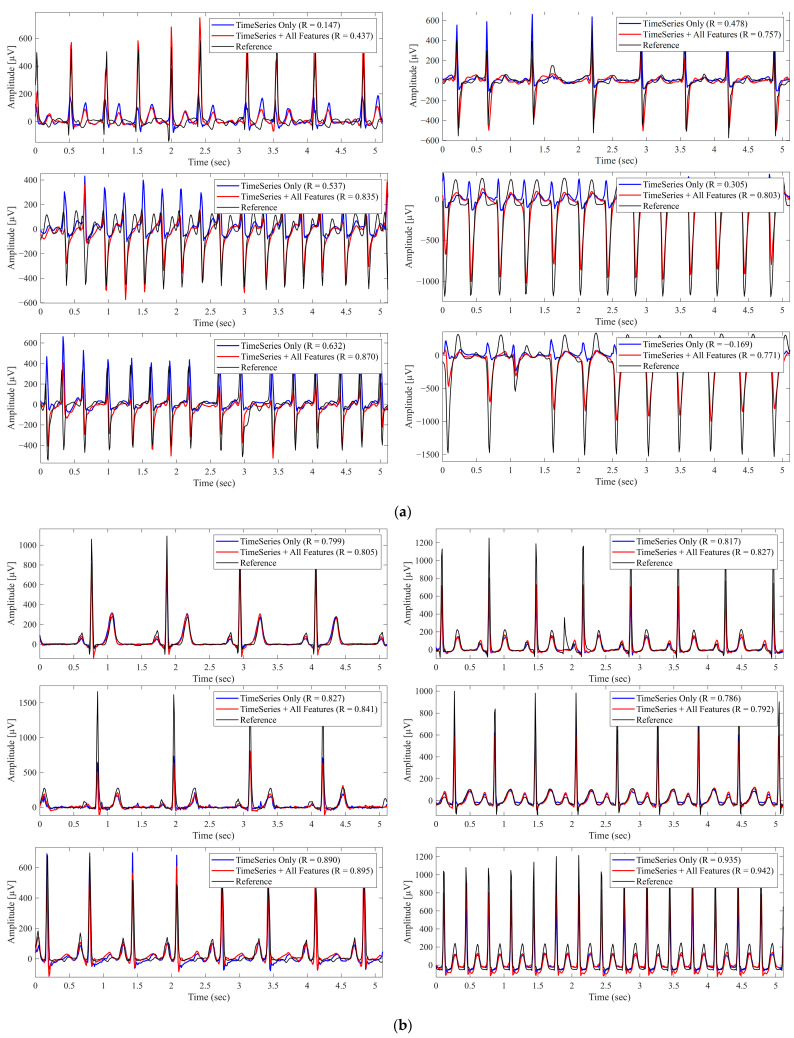
Reconstructed Lead II Waveforms With and Without Metadata Integration. The black lines represent the reference signals, blue lines represent predictions using Lead I alone (Time Series Only), and red lines represent predictions using Lead I combined with all metadata features (Time Series + All Features). ‘R’ denotes the Pearson correlation coefficient. (**a**) Example from a case with morphological abnormalities, where metadata integration improved reconstruction accuracy. (**b**) Example from a different case primarily exhibiting rhythm irregularities, where metadata had minimal impact.

Figure 2 shows representative examples of the reconstructed waveforms obtained using Lead I alone and using Lead I combined with all metadata features, highlighting the improvement achieved through metadata integration. Notably, most cases shown in panel (a) exhibit arrhythmic or morphologically abnormal beats, whereas panel (b) primarily includes beats with rhythm irregularity but without marked morphological abnormalities. These observations suggest that metadata integration yields greater reconstruction benefits, particularly in cases with waveform abnormalities.

### 3.2. Segment-Wise Analysis of Metadata Effects

Table 4 summarizes the segment-wise reconstruction performance when different metadata features were incorporated. The inclusion of RAxis increased the QRS correlation from 0.761 to 0.801, whereas the addition of QRSDuration further improved the correlation to 0.806. These improvements were consistent with the physiological roles of the Raxis and QRS duration in ventricular depolarization. In contrast, the addition of TAxis selectively enhanced the T-wave correlation from 0.668 to 0.721, reflecting its relevance to the ventricular repolarization. When all metadata features were integrated, the overall correlation reached its highest value of 0.811 and 0.725, respectively, and the overall segment-wise correlation improved to 0.677. These results demonstrate that physiologically interpretable metadata selectively reinforces the reconstruction of the corresponding waveform segments, in addition to providing global performance gains.

### 3.3. Comparison with Existing Models

Table 5 summarizes the reconstruction performance of the proposed model and previously reported studies. Although a direct comparison is challenging because of differences in datasets and experimental setups, the proposed model achieved performance metrics competitive with state-of-the-art methods, despite relying on a single lead input. This positions the proposed approach as a viable solution in the current landscape of ECG reconstruction. A strict comparative evaluation was conducted against the U-Net baseline under identical conditions, as detailed in Table 6.

As an ablation study, Table 6 compares the proposed dual-branch model with two baseline models under different input configurations. In the comparison with U-Net, when only time-series input was used, U-Net produced higher correlation coefficients, whereas the RMSE values were comparable between the two models. However, when metadata were incorporated, the proposed model improved both the correlation coefficient and RMSE, whereas U-Net only increased in correlation and exhibited a deterioration in RMSE. Consequently, the proposed method achieved significantly better performance than the two baseline models in all evaluation metrics (R, RMSE, and SSIM). Statistical significance was assessed using the Wilcoxon signed-rank test for correlations and paired *t*-tests for RMSE, with Holm’s correction for multiple comparisons (*p* < 0.05).

### 3.4. Evaluation of Predictive Uncertainty

**Figure 3:**
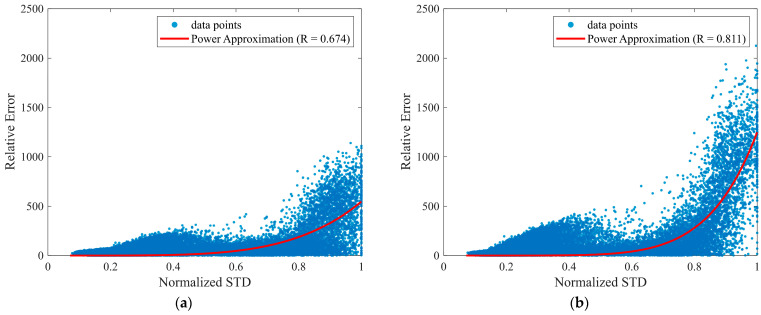
Relationship between predictive uncertainty and reconstruction error. The *x*-axis shows the normalized standard deviation (Normalized STD), defined as $\sigma_{norm}(t)$ in Equation (2), which is used as the predictive uncertainty measure. The y-axis shows the relative error e(t), defined in Equation (3). (**a**) Scatter plot for Lead II; (**b**) Scatter plot for Lead V6.

Figure 3 illustrates the relationship between the predictive uncertainty (standard deviation from the Monte Carlo dropout) and the relative reconstruction error for Lead II, which reveals a moderately positive correlation. Table 7 summarizes the uncertainty–error relationship for each lead using quantile-based analysis of reconstruction errors, indicating a monotonic increase in both relative error and predictive uncertainty with increasing uncertainty quantiles. Table 8 extends this analysis to other leads, where the chest leads generally exhibited stronger correlations, with V6 achieving the highest correlation value. These findings suggest that predictive uncertainty reflects reconstruction error most clearly in diagnostically important chest leads.

**Figure 4:**
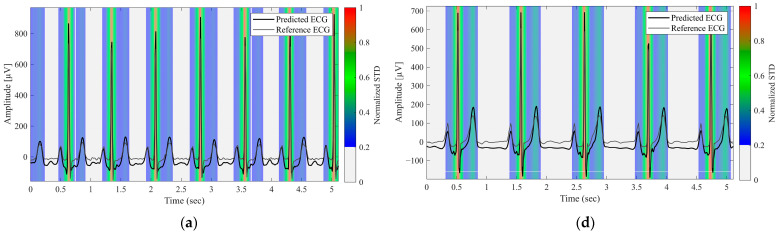

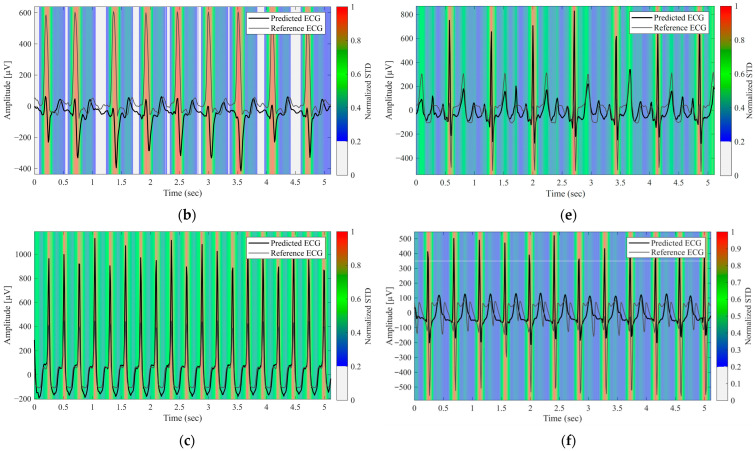
Predictive Uncertainty Heatmaps for Reconstructed Lead II Signals. (**a**) Normal case, (**b**–**e**) Arrhythmic cases with varying waveform abnormalities, (**f**) Case with multiple concurrent abnormalities. The black and gray lines represent the predicted and reference ECG signals, respectively. The background color heatmap represents the predictive uncertainty (normalized STD); red regions indicate high uncertainty (low model confidence), whereas blue regions indicate low uncertainty.

Figure 4 displays the uncertainty heatmaps for the representative normal and arrhythmic cases. Steep waveform regions, such as the R waves, exhibited both higher errors and higher uncertainty, whereas smoother regions, such as the P and T waves, exhibited lower errors and uncertainty. In arrhythmic cases with atypical morphologies, discrepancies between the predicted and reference waveforms coincided with elevated uncertainties. In complex cases with multiple abnormalities, uncertainty remained high across the entire waveforms. These results demonstrate that the proposed approach provides reliability maps that meaningfully reflect waveform complexity and clinical difficulty.

## 4. Discussion

This study introduced a dual-branch deep learning framework that integrates Lead I waveforms with patient metadata for 12-lead ECG reconstruction. The results demonstrated that metadata—particularly axis orientation and conduction intervals—consistently improved the reconstruction performance compared with single-lead input alone (Table 2 and Table 3). Although the magnitude of the improvement was not dramatic, the fact that physiologically interpretable features contributed positively is significant, as it highlights the potential of metadata to enhance both the performance and interpretability. Segment-wise analysis (Table 4) further revealed that metadata exerted selective effects on specific waveform components: RAxis and QRS duration predominantly enhanced the reconstruction of QRS complexes, consistent with their physiological relevance to ventricular depolarization, whereas TAxis primarily improved the reconstruction of T-waves, reflecting its role in ventricular repolarization. Visual inspection of the reconstructed waveforms confirms this quantitative improvement. As shown in the bottom-right panels of Figure 2, the baseline model (time-series only) frequently underestimates the amplitude of steep QRS complexes (specifically the deep downward waves). However, the integration of all metadata enables the model to successfully recover these amplitudes, resulting in a morphology that closely matches the reference. These improvements are clinically meaningful, as QRS abnormalities are associated with conduction disease and bundle branch block [2], whereas T-wave changes are central to the detection of ischemia and repolarization disorders [40].

Furthermore, under metadata-enhanced conditions, the proposed model outperformed the widely used U-Net architecture [20], illustrating that multimodal fusion can provide tangible benefits for ECG reconstruction (Table 6). A comparison with a concatenation-based early-fusion model employing the same CNN–BiLSTM backbone further indicates that late fusion is more effective. With full metadata integration, the proposed dual-branch architecture achieved a higher correlation coefficient (0.792 vs. 0.782) and a lower RMSE (0.111 vs. 0.115 mV), suggesting that the dual-branch design offers more robust integration of heterogeneous modalities. The superior performance of our model, which also extends to the comparison against the widely used U-Net architecture (another early fusion approach), can be quantitatively confirmed by the higher correlation coefficient (0.792 vs. 0.784) and the lower RMSE (0.111 vs. 0.1154 mV) under metadata-enhanced conditions. This is likely because late fusion allows the network to learn high-level waveform representations independently before modulating them with static clinical features, thereby capturing nonlinear interactions more effectively than input-level concatenation. Nevertheless, this finding is based on a limited set of fusion structures and experimental settings, and exploring more diverse fusion designs in future work would further enhance the rigor of the analysis.

Regarding model architecture, recent studies have shown strong performance from alternative deep learning approaches. For example, Srivastava et al. have proposed a GAN-based framework that used a generator–discriminator architecture to reconstruct missing ECG leads from various subsets of available leads [41]. Lan et al. [42] have demonstrated that transformers can achieve high performance in biomedical signal reconstruction, and Kan et al. [43] have shown the effectiveness of graph neural networks for ECG analysis. In this study, we employed a standard CNN–BiLSTM backbone to focus on evaluating the contributions of metadata integration and uncertainty estimation rather than proposing a novel time-series architecture. Notably, the proposed dual-branch framework is backbone-agnostic, enabling the time-series branch to be replaced with more expressive architectures while preserving the proposed fusion design. In the context of the current architecture, a comparison with the U-Net architecture [20] (Table 6) effectively serves as an ablation study, highlighting the necessity and effectiveness of the proposed dual-branch fusion module. These findings are consistent with broader trends in biomedical informatics, where integrating heterogeneous modalities has been shown to enhance predictive performance and facilitate personalized medicine [36,44].

The second key contribution of this study is the incorporation of predictive uncertainty into ECG reconstruction. By applying Monte Carlo dropout [26,27], we obtained temporal variance estimates that correlated with reconstruction errors, particularly in diagnostically important regions such as QRS complexes. This feature offers clinicians an interpretable reliability map that helps identify the waveform regions that warrant closer inspection. Such an approach directly addresses the common criticism that deep learning models operate as “black boxes,” and aligns with broader applications of Bayesian-inspired methods in medical imaging and disease detection [28,29,30,31,32]. Importantly, heatmap analysis (Figure 4) demonstrated that the model tended to assign higher uncertainty to clinically challenging cases, including arrhythmic patterns and atypical morphologies, suggesting that uncertainty estimation can serve as a practical diagnostic aid in wearable ECG systems. Furthermore, lead-specific uncertainty differences provide a quantitative means to assess the reliability of reconstructed 12-lead ECGs, allowing clinicians to identify individual leads that require cautious interpretation. In practice, elevated uncertainty in specific leads may prompt targeted manual review or additional ECG acquisition, supporting safer clinical decision-making.

Although Rajotte et al. [22] recently proposed a multitask CNN framework that combined statement classification with chest lead reconstruction, their approach relied on a reduced subset of five leads (I, II, V1, V3, and V6). Although this strategy achieved competitive diagnostic performance and demonstrated the feasibility of lead reduction, it did not explicitly address predictive uncertainty. In contrast, our study explored a stricter single-lead scenario by using only Lead I supplemented with clinically interpretable metadata. More importantly, we introduced uncertainty estimation via Monte Carlo dropout, which provides reliability maps that highlight waveform regions with reduced confidence. To the best of our knowledge, this is the first study to quantify and visualize predictive uncertainty in ECG reconstruction, thereby complementing reconstruction performance with clinically meaningful interpretability. This distinction positions our framework as a complementary direction to prior studies, emphasizing not only performance but also reliability, which is essential for the real-world clinical deployment of wearable ECG systems.

Further emphasizing the clinical relevance, we found that higher uncertainty values consistently appeared in waveform segments with arrhythmic or morphologically abnormal beats, such as premature ventricular contractions, conduction disturbances, and ST–T abnormalities (Figure 4). These segments also exhibited larger reconstruction errors, indicating that the uncertainty measure appropriately reflects the model’s confidence. From a clinical standpoint, uncertainty information can help identify beats or recordings where the reconstruction is less reliable. Highlighting high-uncertainty regions may support remote monitoring or triage workflows by directing clinicians’ attention to segments that require closer inspection or, when necessary, consideration of a full 12-lead ECG.

This study had several limitations. First, the estimated uncertainty showed a strong correlation with the actual error (r = 0.55–0.81), although the relationship was not perfectly calibrated to be clinically useful. Future studies could explore ensemble methods or variational inference to achieve more robust uncertainty calibration [26,27,28,29,30,31,32]. Second, the model was trained and validated on a single public dataset [33]; external validation across diverse populations and devices is necessary for broader clinical application. Third, the metadata used here were limited to features derivable from Lead I. Incorporating richer metadata, including demographics, comorbidities, or device-specific quality metrics, may provide stronger gains in reconstruction performance. Finally, although this study demonstrated improvements in reconstruction and interpretability, we did not aim to evaluate diagnostic performance. While SSIM values exceeding 0.8 indicate that the proposed method preserves global waveform morphology and temporal structure, the reconstruction accuracy remains insufficient for reliable disease detection. The next important question is whether these benefits can ultimately enhance downstream tasks such as arrhythmia detection, ischemia monitoring [1,2,3,4,5], and other clinically relevant assessments. In particular, reconstructed waveforms can serve as valuable inputs for AI-based diagnostic models [45,46,47,48,49], thereby extending their utility beyond waveform fidelity to clinical applications. Future work will investigate how uncertainty-weighted reconstructed ECGs can improve automated disease classification and clinical decision support, thereby bridging the gap between signal reconstruction and diagnostic reliability.

Overall, the novelty of this study lies in integrating reliability assessment into ECG reconstruction rather than maximizing raw reconstruction performance. By introducing predictive uncertainty into this domain, this study provides a new perspective on how wearable ECG systems can move toward safer, more interpretable, and ultimately more clinically useful applications.

## 5. Conclusions

This study proposed a dual-branch deep learning model that integrates Lead I waveforms with patient metadata for 12-lead ECG reconstruction. While metadata provided only modest performance gains, the proposed model nonetheless outperformed the U-Net baseline under identical experimental conditions. Moreover, the incorporation of Monte Carlo dropout enabled temporal uncertainty estimates that reflected error trends and offered an interpretable measure of model confidence. To the best of our knowledge, this is the first study to apply uncertainty quantification to ECG reconstruction. Overall, the proposed approach enhances the performance, reliability, and interpretability of wearable ECG systems, underscoring their potential as trustworthy diagnostic aids in clinical practice.

## Figures and Tables

**Table 1:** Discretization thresholds for patient metadata.

Feature	Category	Range/Criteria
RAxis/TAxis	Normal	−30°≤θ≤90°
	Left Deviation	−90°<θ<−30°
	Right Deviation	90°<θ≤180°
	Extreme Deviation	−180°<θ≤−90°
QRS Duration	Normal	<120 ms
	Prolonged	≥120 ms
Ventricular Rate	Bradycardia	<60 bpm
	Normal	60–100 bpm
	Tachycardia	<100 ms
QRS Count	Low (Bradycardia)	<10 counts
	Normal	10–16 counts
	High (Tachycardia)	≥17 counts

**Table 2:** Reconstruction performance with individual metadata features.

Input Data	Correlation Coefficients (R)	RMSE [mV]
Time Series Only	0.763 ± 0.134	0.119 ± 0.084
Time Series + RAxis	* 0.787 ± 0.111	* 0.113 ± 0.078
Time Series + TAxis	* 0.788 ± 0.110	* 0.112 ± 0.078
Time Series + VentricularRate	* 0.785 ± 0.113	* 0.114 ± 0.079
Time Series + QRSCount	* 0.788 ± 0.111	* 0.113 ± 0.077
Time Series + QRSDuration	* 0.788 ± 0.110	* 0.112 ± 0.077

* *p* < 0.05 compared to the “Time Series Only” configuration.

**Table 3:** Reconstruction performance with combined metadata features.

Input Data	Correlation Coefficients (R)	RMSE [mV]
Time Series Only	0.763 ± 0.134	0.119 ± 0.084
Time Series + RAxis + QRSDuration	* 0.788 ± 0.111	* 0.112 ± 0.077
Time Series + RAxis + QRSCount	* 0.791 ± 0.109	* 0.111 ± 0.077
Time Series + RAxis + TAxis	* 0.789 ± 0.110	* 0.115 ± 0.078
Time Series + TAxis+ QRSDuration	* 0.788 ± 0.111	* 0.114 ± 0.076
Time Series + TAxis+ QRSCount	* 0.789 ± 0.111	* 0.114 ± 0.078
Time Series + QRSDuration+ QRSCount	* 0.789 ± 0.110	* 0.114 ± 0.078
Time Series + All Features	* 0.792 ± 0.109	* 0.111 ± 0.076

* *p* < 0.05 compared to the “Time Series Only” configuration.

**Table 4:** Segment-wise reconstruction performance with metadata integration.

Input Data	P Wave	QRS Complex	ST Segments	T Wave	Ave
Time Series only	0.571	0.761	0.590	0.668	0.648
Time Series + RAxis	0.575	0.801	0.591	0.672	0.660
Time Series + TAxis	0.574	0.764	0.597	0.721	0.664
Time Series + QRSDuration	0.573	0.806	0.597	0.675	0.663
Time Series + All Features	0.578	0.811	0.593	0.725	0.677

**Table 5:** Comparison of reconstruction performance with previous studies.

Reference	Dataset	Method	ECG Duration (Frequency)	Correlation Coefficients (R)
Hebiguchi et al. [18]	PTB-XL	Bi-LSTM + CNN	5.12 s (100 Hz)	0.78
Chen, Jiarong et al. [38]		MCMA	10 s (500 Hz)	0.77
Savostin, Alexey et al. [20]		U-Net	1.3 s (100 Hz)	0.80
Seo et al. [21]	PTB-XL, SPH&CU DB	U-Net	10 s (500 Hz)	0.76
Zhan et al. [39]	SPH&CU DB, CPSC2018	cGAN	2.05 s (500 Hz)	0.74
Proposed method	SPH&CU DB	Dual Branch (Bi-LSTM + CNN)	5.12 s (100 Hz)	0.79

**Table 6:** Performance comparison between the proposed model and the baseline models.

Model	Time Series	RAxis	Other Features	Correlation Coefficients (R)	RMSE [mV]	SSIM
Proposed Method (Dual Branch: BiLSTM + CNN)	✓			** 0.763 ± 0.134	** 0.119 ± 0.084	** 0.740 ± 0.153
	✓	✓		*^,^** 0.787 ± 0.111	* 0.113 ± 0.078	* 0.796 ± 0.141
	✓	✓	✓	*^,^** 0.792 ± 0.109	*^,^** 0.111 ± 0.076	*^,^** 0.804 ± 0.129
Baseline Method (BiLSTM + CNN)	✓	✓		0.778 ± 0.122	0.116 ± 0.052	0.782 ± 0.149
	✓	✓	✓	0.782 ± 0.121	0.115 ± 0.052	0.785 ± 0.146
Baseline Method (U-Net)	✓			0.771 ± 0.121	0.116 ± 0.079	0.758 ± 0.148
	✓	✓		0.781 ± 0.114	0.114 ± 0.078	0.792 ± 0.146
	✓	✓	✓	0.784 ± 0.113	0.114 ± 0.077	0.795 ± 0.139

✓ indicates that the corresponding feature is used as an input to the model. An asterisk (*) indicates a statistically significant difference from the baseline method (BiLSTM + CNN) under the same input conditions, and a double asterisk (**) indicates a statistically significant difference from the baseline method (U-Net).

**Table 7:** Quantile-based reconstruction errors associated with predictive uncertainty (standard deviation from Monte Carlo dropout) for all ECG data points.

Normalized STD Quantile	Relative Error (RMSE)
Q1	50.8 ± 39.5
Q2	90.7 ± 70.9
Q3	112.5 ± 95.2
Q4	403.0 ± 302.1

**Table 8:** Correlation coefficients between the relative error and predictive uncertainty (standard deviation) across 11 leads.

Lead	Correlation Coefficients (R)
II	0.674
III	0.552
aVr	0.738
aVl	0.642
aVf	0.591
V_1_	0.776
V_2_	0.684
V_3_	0.675
V_4_	0.750
V_5_	0.807
V_6_	0.811

## Data Availability

The ECG data used in this study were provided by Shaoxing People’s Hospital and Chapman University and are publicly available at https://physionet.org/content/ecg-arrhythmia/1.0.0/ (accessed on 25 November 2025). The source code used in this study, including all processing scripts and the complete model implementation required to reproduce the data analysis, is publicly available in the following project repository: https://github.com/kubota0728/multi-lead-ecg-reconstruction (accessed on 9 December 2025). The code is provided without warranty and without an explicit license. For permissions beyond academic or research use, please contact the corresponding author.

## Abbreviations

The following abbreviations are used in this manuscript:

ECG	Electrocardiogram
AI	Artificial Intelligence
CNN	Convolutional Neural Network
RNN	Recurrent Neural Network
LSTM	Long Short-Term Memory
Bi-LSTM	Bidirectional Long Short-Term Memory
1D-CNN	One-Dimensional Convolutional Neural Network
MC	Monte Carlo
RMSE	Root Mean Squared Error
R	Pearson’s Correlation Coefficient
SSIM	Structural Similarity Index
ReLU	Rectified Linear Unit
BPM	Beats Per Minute
RAxis	Right Axis (ventricular depolarization axis)
TAxis	T-wave Axis (ventricular repolarization axis)
Hz	Hertz
s	Seconds
